# Editorial: *Bacillus* and *Pseudomonas* as plant friends: molecular, physiological and ecological interactions

**DOI:** 10.3389/fmicb.2025.1666745

**Published:** 2025-08-12

**Authors:** Tatjana Ćosić, Martin Raspor, Edgardo Jofré, Héctor Herrera

**Affiliations:** ^1^Institute for Biological Research “Siniša Stanković” - National Institute of Republic of Serbia, University of Belgrade, Belgrade, Serbia; ^2^Institute of Environmental Biotechnology and Health, National University of Río Cuarto, Río Cuarto, Córdoba, Argentina; ^3^Departamento de Ciencias Forestales, Facultad de Ciencias Agropecuarias y Medioambiente, Universidad de La Frontera, Temuco, Chile

**Keywords:** plant growth-promoting bacteria, rhizosphere, *Bacillus*, *Pseudomonas*, biological control, cyclic lipopeptides, nutrient solubilization, induced systemic resistance

Today, agricultural crop production is increasingly threatened by climate change worldwide—both directly, e.g., in the form of drought, heat, or floods, and indirectly, by favoring the spread of plant pathogens. The traditional solutions to these problems—synthetic fertilizers and pesticides—that have been shaping the agricultural production over the last 100 years, have proven both insufficient, and ecologically unsustainable in facing these challenges posed by climate change (Schütz et al., 2018).

Over the last decades, increasing attention is being paid to the role of plant growth-promoting rhizobacteria (PGPR) in maintaining soil health and enhancing crop resilience and yield through a diverse palette of symbiotic mechanisms. In particular, the bacteria within the genera *Bacillus* and *Pseudomonas* have proven particularly useful due to their genetic potential that enables them to synthesize an array of molecules that are beneficial to crops, ranging from siderophores and other molecules that help solubilize mineral nutrients thereby reducing the need for chemical fertilization—to lipopeptides, bacteriocins and other metabolites with antibacterial and antifungal activity that efficiently suppress the growth of pathogenic bacteria and fungi (Bhardwaj et al., 2014; Fira et al., 2018; Hashem et al., 2019; Sah et al., 2021).

The aim of this Research Topic was to compile recent research on the beneficial roles of *Bacillus* and *Pseudomonas* species in enhancing plant resilience and growth. The Research Topic features 11 original research articles that demonstrate diverse symbiotic mechanisms between these beneficial bacteria and their host plants. Among these 11 articles, nine focus exclusively on *Bacillus* species (*B. velezensis* strains HY-43, HY-46, R3, Ya-1, BN, TZ01, ZHR0, and LMY3-5; *B. subtilis* strains LK-1, JNF2, and 1JN2; *B. vallismortis* strain SZ-4); one focuses exclusively on two *Pseudomonas* strains (*P. oryzihabitans* D1-104/3 and *P. gessardii* C31-106/3; Popržen et al.) and one article features research on both *B. subtilis* (strain ATCC 6633) and *P. fluorescens* (strain ATCC 17400) in addition to *Azospirillum brasilense* (strain Sp245) (Fourneau et al.). The imbalance in the number of articles featuring the research on *Bacillus* and *Pseudomonas* strains suggests that current research trends in rhizobacterial symbiosis focus notably more on *Bacillus* than on *Pseudomonas* species.

When it comes to the investigated biological roles of these PGPR, it appears that the most widely researched topic is their antifungal action. Five out of the 11 contributions to this Research Topic have investigated the efficacy and underlying mechanisms of *Bacillus* spp. in the biological control of *Fusarium*-induced wilt diseases across a range of crop systems. Thus, Xi et al. reported on the biocontrol effect of LK-1, a *Bacillus subtilis* strain isolated from the rhizosphere soil of wolfberry (*Lycium barbarum*) against *Fusarium oxysporum*. Both the bacterial suspension and the fermentation broth of *B. subtilis* LK-1 demonstrated inhibitory effects on the growth of *F. oxysporum*, whereas the inhibitory effect of the sterile culture filtrate was lower, suggesting a direct inhibitory effect of the living *B. subtilis* LK-1 bacteria on the growth of *F. oxysporum*, in addition to chemical inhibition. Furthermore, wolfberry plants inoculated with the LK-1 fermentation broth demonstrated enhanced growth.

Complementary findings were reported by Yang F. et al., who evaluated a *Bacillus subtilis* isolate (JNF2) obtained from an area affected by *Fusarium* wilt of cucumber (*Cucumis sativus*), caused by *F. oxysporum* f. sp. cucumerinum. The beneficial bacterium exhibited strong antifungal activity and enhanced the growth of cucumber plants. The biocontrol mechanisms involved included biofilm formation, the secretion of antimicrobial metabolites, the induction of systemic resistance in the host plant, and the production of proteases to facilitate plant colonization.

The article by Yang W. et al., which was also conducted in cucumber, demonstrated that inoculation with a *B. subtilis* isolate (1JN2) derived from ginger stems, significantly reduced the severity of *Fusarium* wilt. These effects were linked to changes in root exudate composition post-inoculation, particularly the increased release of amino acids such as glutamine, tryptophan, glycine, and glutamic acid. These changes influenced the rhizosphere microbiome by reshaping its structure and increasing the abundance of beneficial taxa, notably *Hydrogenispora* and *Vicinamibacteria*.

Moreover, the article by Hu et al. analyzed the potential of a *Bacillus vallismortis* isolate (SZ-4) in controlling root rot disease caused by *Fusarium solani* in *Panax ginseng*. The authors report that one of the main mechanisms through which *B. vallismortis* enhances tolerance to *F. solani* is by modulating the structure of the endophytic microbial community. Interestingly, certain co-existing strains, such as *Bacillus velezensis* HY-43 and HY-46, amplified the inhibitory effect against *F. solani* when combined with *B. vallismortis*, suggesting a synergistic interaction.

Similarly, the study by Wang et al. reported how two isolates of *B. velezensis* (R3 and Ya-1), found in a tropical rainforest soil and roots of *Capsicum annuum* L., showed antifungal activity against *Fusarium oxysporum* f. sp. capsici. In addition to effectively suppressing disease, the endophytic strain promoted plant growth, demonstrating its compatibility with the host plant. Furthermore, inoculation modified the rhizosphere microbiota, increasing the relative abundance of beneficial genera, such as *Pseudomonas*, and reducing the presence of *F. oxysporum*.

Among the cyclic lipopeptides produced by *Bacillus* spp., surfactins are recognized as one of the most powerful biosurfactants and exhibit broad-spectrum antimicrobial activity, particularly against phytopathogenic fungi. Because of these properties, surfactins hold considerable potential for biotechnological applications in both the oil industry and agriculture. Moreover, new properties have been attributed to this group of lipopeptides, among them the ability to elicit the plant defense response and to exert cytotoxic effects on cancer cell lines. These emerging functionalities highlight their value as versatile bioactive compounds. Liu et al. provided important insights into the optimization of culture conditions to enhance the production of surfactin in *Bacillus velezensis* BN. Their findings demonstrate that the nitrogen source and amino acid supplementation are key factors influencing surfactin yield. Under optimized conditions, the authors reported a nearly 6-fold increase in production, which was positively correlated with enhanced root colonization of potato plants and antifungal activity against *Phytophthora infestans, Colletotrichum gloeosporioides, Alternaria alternata*, and *Fusarium oxysporum*.

The isolation of new *Bacillus* strains enables the discovery of new lipopeptide variants with unique antimicrobial properties. In this context, An et al. demonstrated that *Bacillus velezensis* TZ01, a strain isolated from the rhizosphere of a citrus tree, exhibited strong antagonistic activity against three major fungal pathogens of citrus. Furthermore, the culture supernatant of *B. velezensis* TZ01 significantly reduced disease severity caused by *Diaporthe citri*. The antifungal activity of strain TZ01 is attributed to the secretion of the lipopeptides surfactin A, bacillomycin D, and fengycin A, which alter fungal cell membranes, leading to growth inhibition. These findings position *B. velezensis* TZ01 as a promising candidate for biological control of citrus fungal diseases.

Similarly, Pan et al. isolated a *Bacillus velezensis* strain, ZHR0, from the phyllosphere of healthy sugarcane plants in an area affected by sugarcane smut (*Sporisorium scitamineum*). The biocontrol efficiency of *B. velezensis* ZHR0 was demonstrated both *in vitro* and *in planta*, including both greenhouse and field-scale trials, with biocontrol efficiency values ranging between 40 and 50%. Whole-genome sequencing of this bacterial strain revealed the presence of gene clusters responsible for the biosynthesis of iturin, fengycin, surfactin, and difficidin, with the LC-MS analysis confirming the production of iturin. Thus, *B. velezensis* ZHR0 can be considered a promising novel biocontrol agent against sugarcane smut.

Finally, Ren et al. used a similar approach in search for biocontrol agents against kiwifruit soft rot (*Botryosphaeria dothidea*): they isolated a bacterial strain LMY3-5 from healthy kiwifruit, and identified it as *Bacillus velezensis*. The cell-free supernatant (CFS) of *B. velezensis* LMY3-5 could inhibit both spore germination and mycelial growth of *B. dothidea*, both in *in vitro* and in *in vivo* assays. Cytological analyses revealed that the fungal cell walls and membranes were degraded when exposed to 16% LMY3-5 CFS. Moreover, the GC/LC-MS analysis of the LMY3-5 CFS revealed the presence of 27 types of small antifungal molecules, suggesting the likely mechanistic explanation of the antifungal action of *B. velezensis* LMY3-5.

In addition to being efficient biocontrol agents against plant pathogens, PGPR are useful plant allies in combatting an array of abiotic stresses. Common duckweed (*Lemna minor* L.) is a suitable model plant for studying the mechanisms of rhizobacterial involvement in plant responses to abiotic stress. Popržen et al. studied the effects of two *Pseudomonas* strains isolated from the duckweed rhizosphere, *P. gessardii* C31-106/3 and *P. oryzihabitans* D1-104/3, on the responses of duckweed to salinity stress. Although both *Pseudomonas* strains showed beneficial effects, these effects were found to differ fundamentally. Namely, the presence of *P. gessardii* C31-106/3 in the duckweed rhizosphere resulted in a slowdown of vegetative growth and switching on adaptive responses to stress such as peroxide signalization. On the other hand, *P. oryzihabitans* D1-104/3 favored a partial adaptive response along with maintaining growth rates, which resulted in visible chlorosis. The results of this work illustrate the complexity and diversity of mechanisms through which rhizobacteria enhance stress tolerance in plants, even among species within the same genus, *Pseudomonas*.

Despite the numerous benefits of PGPR, their actual efficiency as biofertilizers is affected by an array of environmental factors. Among them, the biocompatibility between the host plant and its rhizobacterial symbiont play an important role in the establishment of efficient rhizosymbiotic relationships. Fourneau et al. demonstrated that not all host plant-PGPR species pairs are equally efficient at establishing symbiosis, which they quantified through parameters such as bacterial chemotactic responses to root exudates, and bacterial generation time and growth rates on media containing root exudates. The results showed that generally, the root exudates of oilseed rape (*Brassica napus*) were more efficient at attracting and feeding the PGPR than those of pea (*Pisum sativum*) and ryegrass (*Lolium perenne*); however, pea root exudates were the most efficient at stimulating bacterial biomass. On the other hand, *Pseudomonas fluorescens* and *Azospirillum brasilense* showed a general tendency to respond more readily to the root exudates of all the three plant species, than *Bacillus subtilis* did. The findings of this study thus suggest that compatibility between the host plant and its rhizoinoculants must be considered for maximum output when planning the application of biofertilizers in the field.

In conclusion, this Research Topic presents a collection of current original research articles dedicated to the beneficial roles of *Bacillus* and *Pseudomonas* bacteria as plant symbionts, revealing the mechanisms of their biocontrol activity against fungal pathogens, but also highlighting the diversity and versatility of their effects on plant tolerance to abiotic stress, while at the same time calling for tailored pairing of beneficial PGPR strains with their target crop plants for enhancing the viability and efficiency of the plant-PGPR symbiosis. The discovery, isolation, and identification of novel PGPR strains, notably of the *Bacillus* and *Pseudomonas* genera from novel plant sources, along with latest technological tools such as genomics, metabolomics, multi-omics for beneficial metabolite detection and identification, or design-of-experiment (DoE) approaches for optimization of biotechnological production of these metabolites, hold promise for a more sustainable future in which the use of synthetic fertilizers and pesticides will gradually decline thanks to our capability to make the most of the biological potential of these precious tiny allies.
